# Electroencephalographic Features of Presumed Hepatic Encephalopathy in a Pediatric Dog with a Portosystemic Shunt—A Case Report

**DOI:** 10.3390/life15010107

**Published:** 2025-01-16

**Authors:** Raluca Adriana Ștefănescu, Vasile Boghian, Gheorghe Solcan, Mario Darius Codreanu, Mihai Musteata

**Affiliations:** 1 Neurology Service, Faculty of Veterinary Medicine, “Ion Ionescu de la Brad” Iași University of Life Sciences, 700489 Iași, Romania; raluca.stef@yahoo.ro (R.A.Ș.); mihai.musteata@iuls.ro (M.M.); 2 Internal Medicine Clinic, Faculty of Veterinary Medicine, “Ion Ionescu de la Brad” Iași University of Life Sciences, 8 M. Sadoveanu Alley, 700489 Iași, Romania; 3 Internal Medicine Clinic, Faculty of Veterinary Medicine, University of Agronomical Sciences and Veterinary Medicine, 105 Independency Spl., 050097 Bucharest, Romania; codveterinary@yahoo.com

**Keywords:** dog, portosystemic shunt, seizures, computed tomography, electroencephalography

## Abstract

Hepatic encephalopathy (HE) in dogs is a metabolic disorder of the central nervous system that occurs secondarily to liver dysfunctions, whether due to acquired or congenital causes. A portosystemic shunt is the presence of abnormal communications between the hepatic vessels (portal and suprahepatic veins). As a result of this, the blood brought from the digestive tract through the portal vein bypasses the liver, and the unmetabolized components of the portal bloodstream enter directly into systemic circulation, causing clinical symptoms of metabolic encephalopathy (HE). A 3-month-old Bichon canine patient with a history of seizures secondarily to a portosystemic shunt (PS), confirmed through color Doppler ultrasound exam and computed tomography, was presented for evaluation. The typical electroencephalographic (EEG) traces recorded were characterized by the presence of bilateral symmetrical triphasic waves, resembling non-convulsive status epilepticus. The presence of this EEG pattern is useful in choosing the best therapeutic option in order to not accentuate the HE sings and, consequently, to decrease the mortality risk due to a prolonged status epilepticus.

## 1. Introduction

Hepatic encephalopathy (HE) in dogs is a metabolic disorder of the central nervous system that occurs secondarily to liver dysfunction, regardless of whether they have acquired or congenital causes [1,2,3]. One of the most common causes of HE is the presence of abnormal communications between hepatic vessels (portal vein and suprahepatic vein) that form a portosystemic shunt. As a result of this, the blood brought from the digestive tract through the portal vein bypasses the liver, and the unmetabolized components of the portal bloodstream enter directly into the systemic circulation, causing clinical symptoms of metabolic encephalopathy (ME). Toxic substances, such as ammonia, aromatic amino acids (including phenylalanine, tyrosine, and tryptophan), short-chain fatty acids, mercaptans, and various biogenic amines, indoles, and skatoles have been implicated in causing HE. These compounds have convulsive effects in animals, and can modulate cortical excitability by acting as GABA-A receptor antagonists and NMDA receptor agonists, thus leading to an increase in cortical excitability [4]. Metabolic encephalopathy due to portosystemic shunting causes functional disturbances of CNS activity due to endogenous intoxication.

Secondary hepatic encephalopathy (HE) due to portosystemic shunting is easily suspected if clinical signs appear following food ingestion. Historically, it has been accepted that neurological symptoms manifest 30–90 min after ingestion, and disappear within a few hours, with seizures being the most expressive clinical signs [3]. Suggestive clinical signs, breed predisposition, and additional tests (laboratory tests, along with routine imaging such as ultrasound, or advanced techniques such as computed tomography, etc.) are sufficient to establish the diagnosis of vascular anomaly [4,5]. In reality, neurological clinical signs are present continuously, but if their intensity is very low, the clinical signs may be subtle and expressed only through mild, nonspecific cortical inhibition or changes in behavior [4]. Especially in those animals, assessing the neurological impact of this vascular anomaly is challenging.

Electroencephalographic (EEG) examination is a procedure that allows the real-time assessment of the bioelectric activity of the cerebral cortex [6,7,8] and the occurrence of superimposed generalized spikes or sharp waves on a slow wave background is a common finding [9]. In humans, understanding the specific EEG patterns in patients with portosystemic shunting is helpful for clinicians in establishing a diagnosis, assessing the severity of cerebral homeostasis disruption, and monitoring the therapeutic effects of medication administered to patients up to the time of surgery (if feasible) [10]. In early stages of HE, EEG traces are characterized by an increase only of beta activity [11] or alpha activity [12] into the frontal lobes, evolving in more severe stages of HE, to bioelectric transients as triphasic waves and bursts of intermittent rhythmic delta activity are seen both in dogs and humans [8,10].

Rapid identification of the etiology in a seizuring or unconscious dog and specific treatment initiation should be rapidly performed, as long as a prolonged unconscious status is associated with a poor outcome [13,14]. This goal may be difficult to achieve, especially in patients with structural brain events who needs advance imaging equipment to be diagnosed, or in patients with nonconvulsive status epilepticus due to either a systemic metabolic disease or a primary structural brain lesion. In such scenarios, identification of a specific pattern of the bioelectric cerebral activity together with routine lab results might offer the clinician a quick indication over both the etiology of the encephalopathies and also the patient’s prognosis and potential treatment efficacy.

The aim of the present study is to present the typical EEG recorded in a pediatric canine patient with a presumed HE secondarily to an extrahepatic PS.

## 2. Case Description

An intact, crossbred male Bichon Frisé, 3 months old and weighing 2 kg, was admitted to the Neurology Service of the Veterinary Teaching Hospital of FMV, Iași, for evaluation of epileptiform seizures with a 4-day history. Seizure activity was characterized by loss of consciousness, rolling and pedaling movements, hypersalivation, and spontaneous emission of urine, which occurred mainly postprandial. Upon admission, the patient was presented with an inconstant opisthotonus with thoracic limb extension and pelvic limb flexion beneath the body (mimicking a decerebellate rigidity), along with severe cranial nerve deficits: normal blink reflex, but delayed and incomplete photomotor reflex, delayed physiological nystagmus, bilateral ventral positional strabismus, bilateral absent menace response, and an absent response to bilateral nasal cantus stimulation. A diffuse intracranial multifocal condition (forebrain, brainstem, and potentially cerebellum) was suspected, and a metabolic, toxic, inflammatory etiology was privileged.

**Figure 1:**
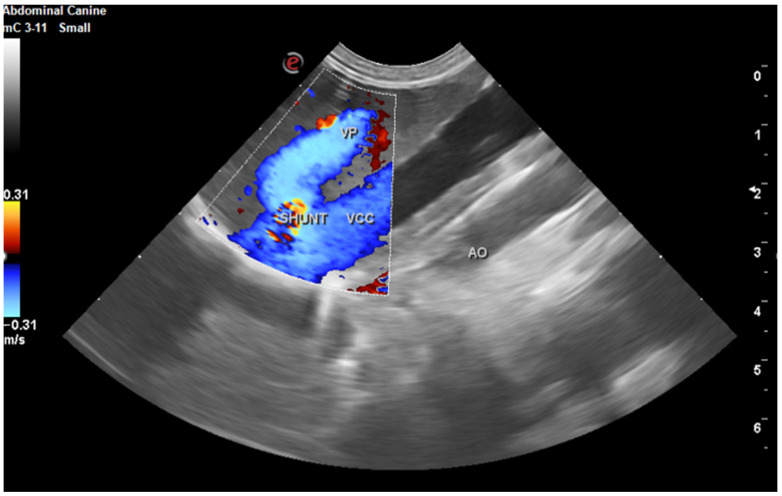
Abdominal ultrasound exam, with color Doppler reveal portocaval shunt. VP = portal vein. VCC = caudal cava vein. AO = abdominal aorta.

**Figure 2:**
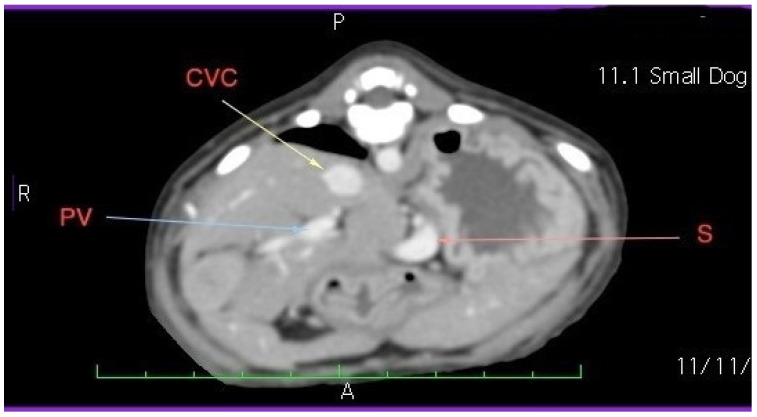
Abdominal CT exam. Large extrahepatic portocaval shunt, Bichon dog, 3 months. Following the caudal vena cava (CVC) from the caudocranial direction, it is observed that it runs parallel to the portal vein to the prerenal level (cranial to the kidneys), where it gives a ventral branch that descends ventro-laterally to the left, follows the small curvature of the stomach, then turns to the right and joins the portal vein (PV) at the level of the hepatic hilum.

**Figure 3:**
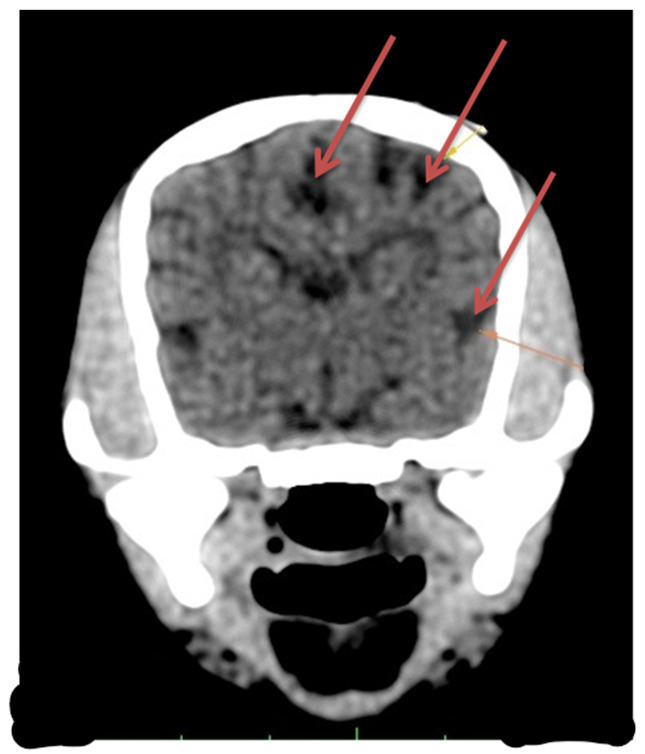
Cerebral CT exam in a Bichon dog, 3 months old, with portocaval shunt. Multiple hypoattenuated lesions with little to no contrast enhancement, some located in the cortex with a conical shape, others more extensive and biconvex, as well as along the cerebral sulci.

Laboratory tests showed a hemogram with neutrophilic leukocytosis, and serum biochemistry revealed abnormalities in routine hepatic markers, including AST 130 U/L (normal range of 14–45 U/L), ALP 207 (normal range of 20–150 U/L), and GGT 9 (normal range of 0–7 U/L). An abdominal ultrasound examination (My Lab 40 ultrasound machine, Esaote Pie Medical, Maastricht, The Netherlands) revealed a congenital portosystemic shunt (Figure 1) (in comparison to a physiological aspect, Figure S1), for which the patient was recommended to undergo bile acid testing and advanced imaging (abdominal CT). For a rapid diagnosis and due to financial reasons, the owner agreed to advanced imaging, and temporarily declined the bile acid testing. CT examination under chemical restraint (Ketamine, Kepro, Deventer, The Netherlands, at 0.1 mg/kg IV and medetomidine, Domitor, Orion Pharma, at 0.05 mg/kg IV) revealed a large extrahepatic porto-caval shunt (Figure 2) associated with multiple conical cortical brain lesions that were hypodense, with reduced or absent contrast uptake, along with more extensive biconvex lesions, including along the cerebral sulci (Figure 3). A final diagnosis of portosystemic shunt and presumed hepatic encephalopathy was established.

**Figure 4:**
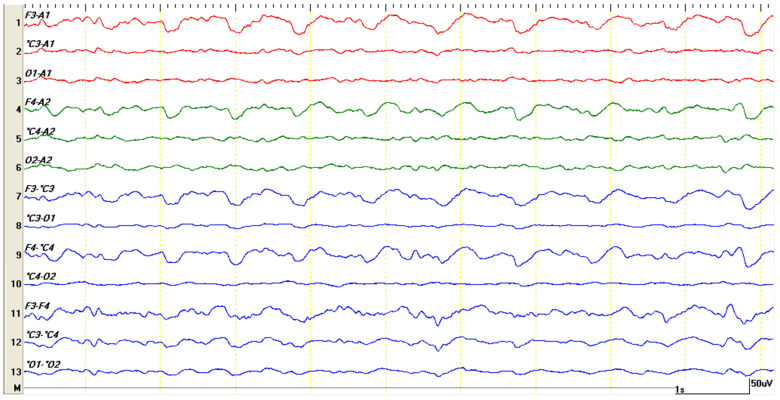
EEG recording for 10 s. The nearly normal cerebral activity of the patient immediately after the CT study, while still under medetomidine–ketamine anesthesia, showed slow, symmetric background activity characterized by delta waves (with a frequency of 1–4 Hz). Each second of the recording is delimited by the yellow vertical markings.

**Figure 5:**
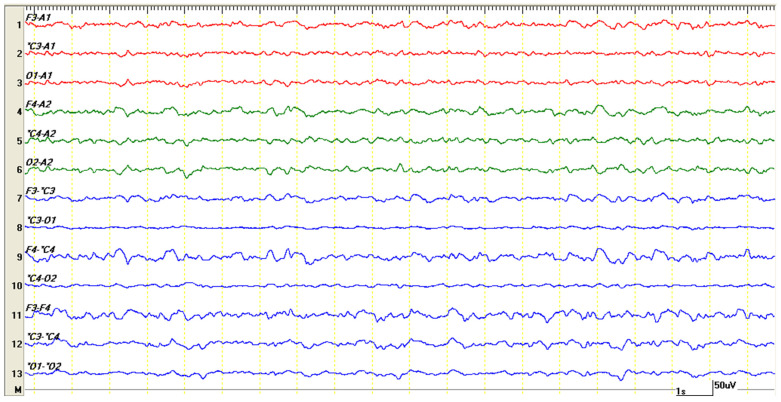
Slow background rhythm, theta (same patient) appears as the effects of anesthesia from the CT examination wear off. Compared to the previous image, a gradual increase in frequency and amplitude can be observed.

Following the CT study, under the same anesthesia, the bioelectrical activity of the brain was recorded using a Neurofax electroencephalograph (Nihon Kohden). Nine needle-type electrodes were placed as follows: F3 (left frontal), F4 (right frontal), C3 (left center), C4 (right center), O1 (left occipital), O2 (right occipital), A1 (left ear), A2 (right ear), and the ground Z electrode (positioned on the muzzle above the nose). Recordings were made in both a referential montage (F3-A1, C3-A1, O1-A1, F4-A2, C4-A2, and O2-A2) and a bipolar one (F3-C3, C3-O1, F4-C4, C4-O2, F3-F4, C3-C4, and O1-O2). The electroencephalographic recording was conducted using the following parameters: a sensitivity of 75 μV, a time constant of 0.3 s, a filter pass-down of 70 Hz, a filter pass-up of 30 Hz, and electrode impedance <10 Ω. The electrical activity of the brain was characterized by almost normal waves, represented by slow, symmetrical, and predominantly delta-wave background activity (Figure 4). The delta rhythm is commonly found in deep sleep or during anesthesia. In the case of anesthesia, once its effect ends, background activity is characterized by the appearance of a high-frequency waves that gradually pass through theta rhythms (Figure 5) to alpha and beta rhythms, with the latter marking the onset of waking status.

**Figure 6:**
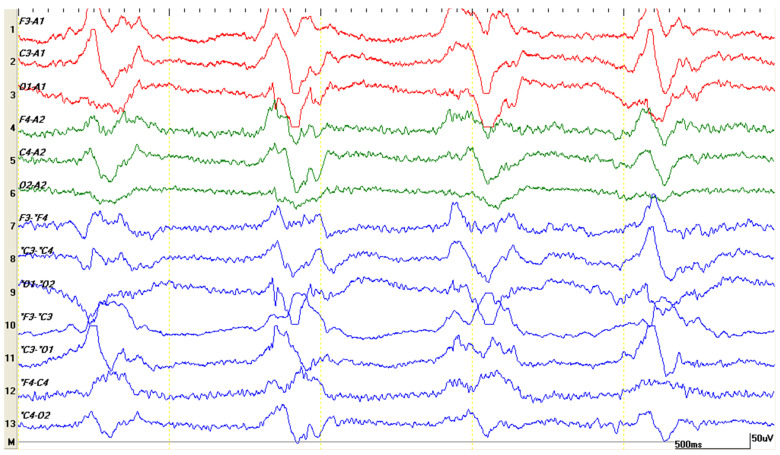
Periodic discharges with low frequency but high amplitude, reaching and exceeding 150 microvolts. Clinically, the patient exhibited paddling movements and rhythmic vocalizations synchronized with the graphoelements.

**Figure 7:**
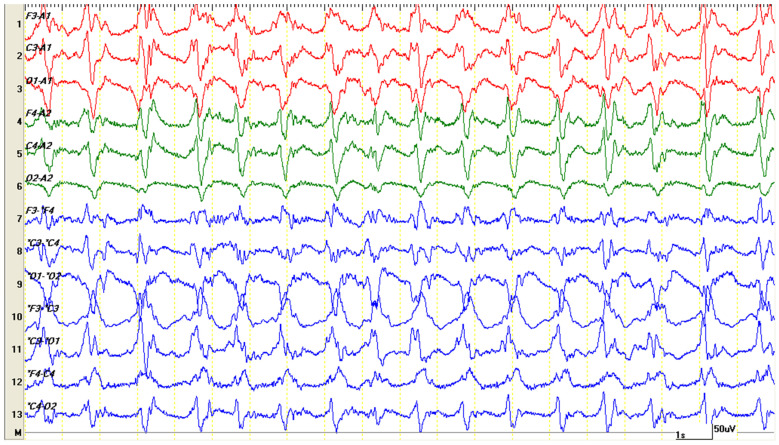
This figure displays a 20 s segment, compressed from the previous EEG trace, revealing the hypersynchronization of cerebral electrical activity. The primary graphoelements, which may resemble spikes, consist of sharp and slow wave complexes with three distinct phases, known as “triphasic waves”.

**Figure 8:**
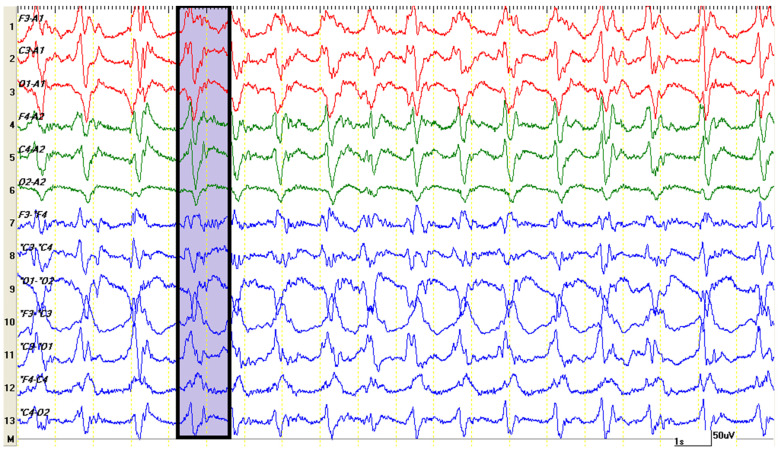
The morphology of bilateral symmetric triphasic waves is characterized by a high amplitude (exceeding 70 µV) and consists of three distinct phases: an initial small negative deflection, a prominent positive deflection, and a prolonged, slow negative deflection that gradually increases in amplitude. These waves exhibit a diffuse and bilaterally synchronous distribution.

In the case of the described patient, a few minutes after the onset of the theta rhythm, the appearance of periodic, rhythmic, low-frequency discharges and a very high amplitude (over 150 microvolts) was observed on the EEG traces. Graphoelements could be identified in all leads, both in mono- and bipolar montages in a synchronous clinical context, represented by the installation of pedaling movements and rhythmic vocalizations (Figure 6). In addition to the hypersynchronized cerebral electrical activity, “bilateral triphasic waves” (Figure 7) were identified. These were characterized by complexes with a moderate to high amplitude (100–300 μV) of a frequency of 1.5 to 5 Hz (Figure 8). Although they were frequently prevalent in the frontal regions, they could also be seen in the occipital channels. The initial negative component was the sharpest, while the next positive part of the complex was the largest and subsequently followed by another negative wave. Persistent asymmetry (that is not related to technical factors or a cranial defect) may suggest an underlying structural injury on the side with the lower amplitude.

After presenting the diagnosis and treatment options, the owners opted for euthanasia.

## 3. Discussion

In veterinary practice, EEG is commonly used for confirming the diagnostic of idiopathic epilepsy (Tier III according to the International Veterinary Epilepsy Taskforce consensus [13]). Over the last few years, the availability of EEG equipment increased and a large number of studies covering both the technical standardization and the findings in different encephalopathies were published [15,16,17,18]. According to Luca et al. (2023), other EEG indications besides diagnosing epilepsy, identification of epileptic foci, and antiepileptic drug efficacy include determining brain death, sleep disorders, research purposes, and post-op brain surgery monitoring [19]. In this paper, we describe the EEG findings in a pediatric dog with presumed HE due to an extrahepatic portosystemic shunt.

Portosystemic shunt (PS) is one of the most common congenital liver diseases in dogs [20]. Clinical signs associated with PS in dogs are various and involve neurological, digestive, and urinary system. Neurological signs are the expression of the hepatic encephalopathy (HE), and may vary for very subtle one (i.e., apathy) to severe ones (seizure, coma) [2]. Despite the fact that PS is easily diagnosed when the blood tests and imaging results are performed, HE diagnosis may be challenging for the veterinarian. Several studies performed both in humans and dogs show that EEG may be considered an useful diagnostic tool in diagnosing (and evaluation) of HE, as long as it can be associated with the presence of bilateral symmetric triphasic waves, a characteristic bioelectric pattern. The morphology of bilateral symmetric triphasic waves is characterized by a high amplitude (>70 µV) and consisting of an initial small negative deflection, followed by a large positive deflection and a long, slow, extended negative deflection that gradually increases, with a diffuse and bilaterally synchronous distribution [21,22,23]. In our dog, immediately after the diagnostic of PS was fixed by both ultrasound examination and CT exam, under the same anesthesia, the EEG traces show the presence of bilateral symmetric triphasic waves.

In humans, other potential causes for the occurrence of bilateral symmetric triphasic waves include focal structural lesions and concomitant toxic or metabolic encephalopathy (hyperthyroidism, hypo/hyperglycemia, hypo/hypercalcemia, thyamine deficiency, etc.) [14,21,22]. In veterinary patients, the presence of EEG periodic discharges was observed beside HE secondarily to PS in seizuring dogs due to a wide variety of etiologies, both intracranial and systemic, during the preictal, ictal or postictal period making their interpretation challenging [15]. Specifically, according to the same research group, triphasic waves were noticed in relationship with the presence of intra-axial mases, polycythemia and idiopathic epilepsy [15]. In our dog, based on the clinical symptomatology, blood tests and imaging results, we suspect that the occurrence of the bilateral triphasic waves were directly related with the development of HE. In a study conducted on 12 dogs with hepatic encephalopathy (due to portosystemic shunt) the EEG recordings revealed generalized high-voltage, low-frequency bioelectric activity, with the presence of symmetrical bilateral triphasic waves in 58% of cases [24]. In humans with HE, the presence of triphasic waves is a marker of severe HE (potentially as a result of white matter edema) and is a and indirect marker of increased risk of death [25].

In dogs, the occurrence of HE was historically associated with high ammonia levels, and some researchers state that the occurrence of triphasic waves in patients with HE is directly dependent on the levels of ammonia in systemic circulation [25]. However, both old and recent studies underline the idea that the HE may appear even in patients with a normal blood ammonia level. Moreover, in humans, the fasting ammonia concentration is nowadays considered an unreliable indicator of the degree of encephalopathy [26] and in dogs, the correlations between blood ammonia concentrations and disease severity (i.e., hepatic encephalopathy grade) were rather weak for dogs with extrahepatic portosystemic shunt [27]. Even though the absence of ammonia determination may be considered as a potential limitation in HE diagnosis in our dog, the clinical symptomatology and the imaging data were highly suggestive for a severe HE diagnostic.

At the time of presentation, the history (seizures) and cranial nerve deficits suggested a neurological localization at the level of the forebrain, with profound disruption of neuronal homeostasis in this area demonstrated by the electrical activity recorded in the EEG. However, it is interesting that, at its presentation, the patient exhibited decerebellate rigidity, suggesting a cerebellar lesion (and possibly an edema with “mass phenomenon”). The EEG technique does not record the activity of cerebellar neurons; therefore, we could not assume that an electrical alteration in the brain was necessarily present in the cerebellum as well. Furthermore, CT examination is not the preferred method for investigating cerebellar parenchyma in small animals. Magnetic resonance imaging (MRI) is a more suitable method for investigating the cerebellum, which represents a limitation in our study [28]. However, according to Weiss and Thabut [29], CT scans are still useful for exploring the pathophysiology of HE, whereas brain MRIs are cumbersome to perform in critically ill patients, and frequently not easily available. Also, the authors underline that for hepatic encephalopathy, CT scans could give some surrogate markers for evaluating treatment strategies [29]. In rats with experimentally induced portosystemic shunts, the morphological evidence demonstrated, in addition to HE, cerebellar impairment, including Purkinje cell loss, astrocyte and Bergmann glial cell swelling, microglial activation, and the onset of cytotoxic edema [30]. In dogs, congenital portosystemic shunting was associated with marked cellular changes in both cerebral cortex and cerebellum [31]. We suspect that the decerebellate rigidity may have been associated with cerebellar edema, which may have been of a higher grade than the compensated cerebral edema.

Another potential limitation of our study could be that total bile acid measurement were not performed. Bile acids test is a helpful marker in diagnosing the portosystemic shunt (and not the hepatic encephalopathy). The test’s diagnostic sensitivity and specificity range from 88% to 93% and from 68% to 87%, respectively [32,33]. As a consequence, based only on bile acids test a diagnostic of portosystemic shunt (even if the test is a positive one) is only suspected but not confirmed. The advance imaging (as with computer tomography and Doppler ultrasonography) are preferred for establishing a final diagnosis [2]. In our manuscript, we proved the shunt by both imaging examinations: CT examination and Doppler ultrasonography. As a consequence, in this light, we consider that the existence of the shunt was proved by more accurate techniques (and not only suspected based on a potential bile acids test).

In the case of the presented patient, upon emergence from anesthesia, the EEG trace was characterized by continuous abnormal hypersynchronous activity, similar to non-convulsive status epilepticus (Figure 8). Triphasic waves are not epileptiform per se, and are not usually associated with seizures. However, generalized triphasic waves can resemble the pattern of non-convulsive status epilepticus. In the few studies conducted in human medicine, differentiating EEG traces between these two entities is difficult, and the fact that both resolve after the administration of diazepam may lead to diagnostic confusion [34,35]. In our case, although the patient had a history of seizures, the recorded EEG traces could not be definitively classified as non-convulsive status epilepticus. In the case of uncertainty, the application of an emergency anti-convulsant medication is preferred. However, despite benzodiazepine being considered the first line medication in treating status epilepticus in dogs [36], the use of benzodiazepine for stopping status epilepticus in dogs with HE due to PS may be controversial as long as no clinical trials exist in veterinary medicine, and in humans, their administration might precipitate the HE [2]. In such situation levetiracetam is considered to be a safer drug in achieving the therapeutic goal. In this light, in dogs with suspected HE secondarily to PS and triphasic waves on EEG recordings the use of EEG might be offer a rapid treatment orientation without increasing the outcome risks. As we previously mentioned, in humans, the triphasic waves pattern occurs especially in 2nd to 3rd grade HE [10]. Hence, repeated EEG recordings in dogs with HE may be useful for the objective assessment of the recovering the brain functionality and indirect the therapeutic efficacy of PS in dogs.

## 4. Conclusions

In this study, we describe a pediatric canine patient with reactive epileptic seizures following a presumed hepatic encephalopathy due to an extrahepatic portosystemic shunt. The EEG trace was characterized by the presence of bilateral synchronous three-phase wave bursts, resembling non-convulsive status epilepticus. EEG can complement the diagnosis of portosystemic shunting and provide an objective picture of the change in cerebral electrical homeostasis and might be useful in initiating the therapeutic protocol.

## Data Availability

The data presented in this study are available in the manuscript.

## Supplementary Materials

The following supporting information can be downloaded at: https://www.mdpi.com/article/10.3390/life15010107/s1, Figure S1a. Abdominal ultrasound exam of the liver in healthy dog, M mode, reveal distinct traject of portal vein (VC) and caudal cava vein (VCC). Figure S1b. Abdominal ultrasound exam of the liver in healthy dog, Doppler mode, reveal distinct traject of portal vein (VC) and caudal cava vein (CVV).
