# Supplementary figures and images for: Electroencephalographic Features of Presumed Hepatic Encephalopathy in a Pediatric Dog with a Portosystemic Shunt—A Case Report
Source: Life (Basel). 2025 Jan 16;15(1):107. doi: 10.3390/life15010107 (PMC11767108; doi:10.3390/life15010107)

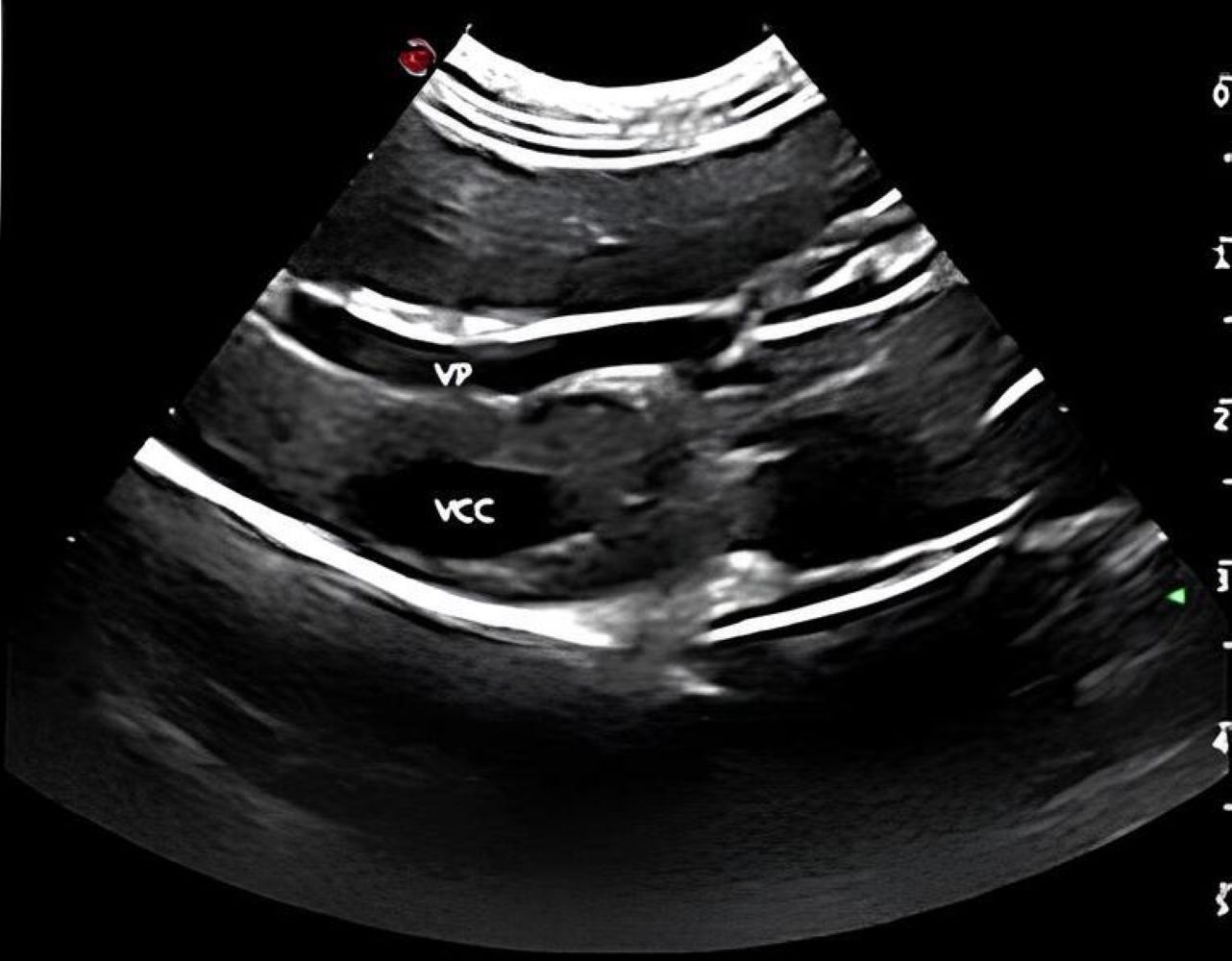

Supplement: Supplementary file 1 [file life-15-00107-s001.zip › figure S1/Figure S1 a.jpg]

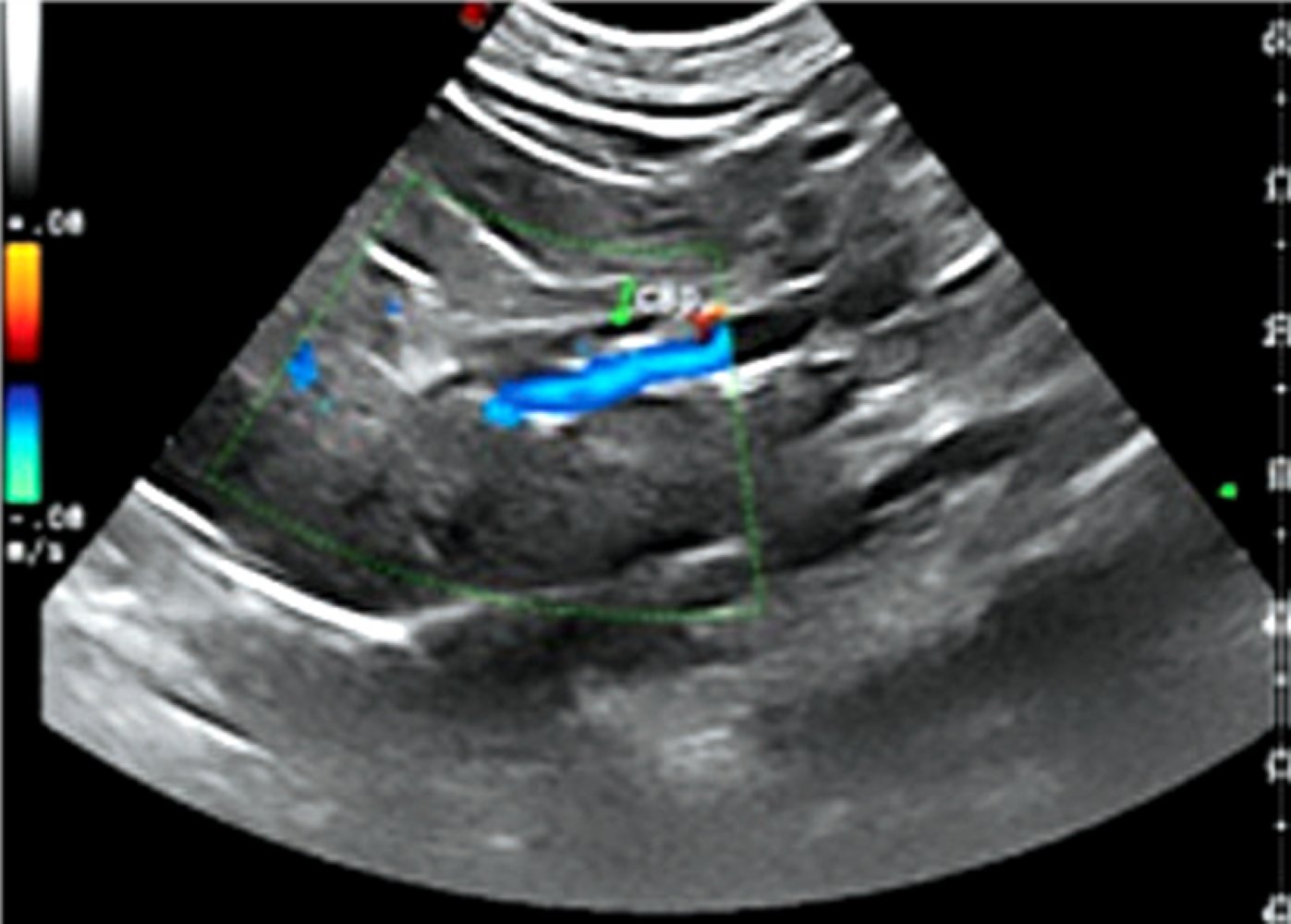

Supplement: Supplementary file 1 [file life-15-00107-s001.zip › figure S1/Figure S1 b.png]
